# Role of carbohydrate moiety in immunoreactivity of Ovomucoid

**DOI:** 10.1186/2045-7022-3-S3-O13

**Published:** 2013-07-25

**Authors:** S Benedé, I López Expósito, E Molina, R López-Fandiñ

**Affiliations:** 1 Bioactivity and Food Analysis, Institute for Food Science Research (CIAL) (CSIC-UAM), Madrid, Spain

## Background

An important question pertaining to glycoproteins is whether the covalently-bound carbohydrate moieties contribute to its immunogenic activity. Ovomucoid (OM) is, for these kinds of studies, a model compound in view of its relatively high carbohydrate content but the role of the carbohydrate moieties on its immunogenic activity still remains controversial. The aim of this study was to establish the effect of glycosylation on the IgE-binding properties and biological activity of OM.

## Methods

Carbohydrate moieties of OM were removed by enzymatic deglycosylation with PNGase F and the degree of deglycosylation of the protein was evaluated by SDS-PAGE with PAS staining and RP-HPLC. Changes in secondary and tertiary structure of OM due to deglycosylation were evaluated by circular dichroism. Immunoreactivity of native and deglycosylated OM was evaluated by inhibition ELISA and western blot using the sera from ten egg-allergic patients with IgE levels from 37 to more than 100 kU/L. Using PBMCs from egg-allergic patients, the cellular proliferative potency and the capacity to stimulate the secretion of interleukins of native and deglycosylated OM were assessed. Basophil activation by OM, with and without carbohydrates, was assessed using human basophils passively sensitized with serum from children with egg allergy.

## Results

PNGase F effectively released carbohydrates attached to OM after five days at 37° without apparent structural changes. Immunoassays showed that, in eight out of ten sera of egg-allergic patients, the IgE binding to deglycosylated OM was less effective than to the native protein. The results of immunoblotting showed that six out of ten sera, previously incubated with the deglycosylated protein, kept their IgE binding capacity to the glycosylated protein, supporting the idea that carbohydrates may play a role in antigen recognition. In spite of these results, no influence of glycosylation on T cell activation and proliferation was found.

## Conclusion

The carbohydrate chains attached to OM play a role in the reactivity against IgE, but their clinical relevance might be low.

## Disclosure of interest

None declared.

